# From element to development: the power of the essential micronutrient boron to shape morphological processes in plants

**DOI:** 10.1093/jxb/eraa042

**Published:** 2020-01-27

**Authors:** Michaela S Matthes, Janlo M Robil, Paula McSteen

**Affiliations:** 1 Division of Biological Sciences, Bond Life Sciences Center, Interdisciplinary Plant Group, and Missouri Maize Center, University of Missouri, LSC, Columbia, MO, USA; 2 University of Missouri, USA

**Keywords:** Arabidopsis, BOR1, boron deficiency, boron transport, boronic acids, maize, NIP5, 1, phenylboronic acid, rice, *rottenear*, *tassel-less1*

## Abstract

Deficiency of the essential nutrient boron (B) in the soil is one of the most widespread micronutrient deficiencies worldwide, leading to developmental defects in root and shoot tissues of plants, and severe yield reductions in many crops. Despite this agricultural importance, the underlying mechanisms of how B shapes plant developmental and morphological processes are still not unequivocally understood in detail. This review evaluates experimental approaches that address our current understanding of how B influences plant morphological processes by focusing on developmental defects observed under B deficiency. We assess what is known about mechanisms that control B homeostasis and specifically highlight: (i) limitations in the methodology that is used to induce B deficiency; (ii) differences between mutant phenotypes and normal plants grown under B deficiency; and (iii) recent research on analyzing interactions between B and phytohormones. Our analysis highlights the need for standardized methodology to evaluate the roles of B in the cell wall versus other parts of the cell.

## Teaser

We highlight different approaches used to induce boron deficiency in plants and how this has led to differences in interpretation of boron’s role in the cell wall versus the cytosol.

## Introduction

**Fig. 1.**
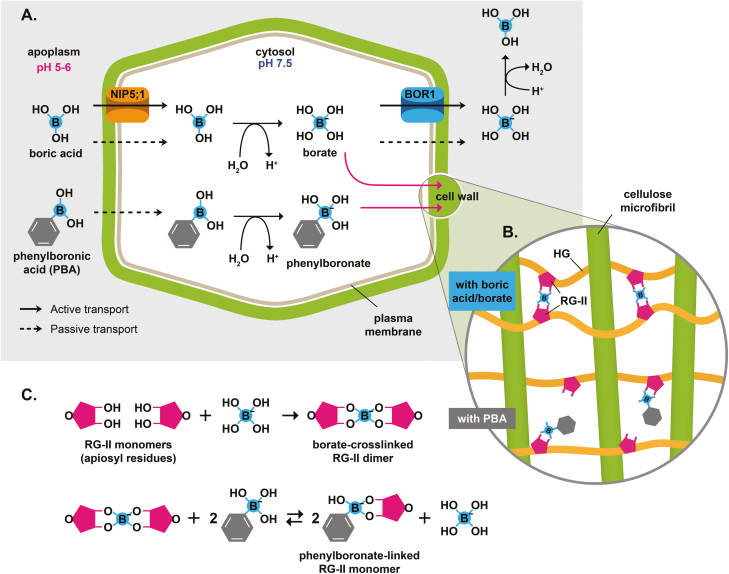
Boric acid (BA)/phenylboronic acid (PBA) uptake into plant cells. (A) Passive and active uptake mechanisms of BA and PBA through either diffusion or the NIP5;1 channel protein. Due to the cytosolic pH, BA and PBA are diverted into borate and phenylboronate anions, which react with the apiosyl residues of rhamnogalacturonan-II (RG-II) in the cell wall. Export of borate and phenylboronate out of a plant cell occurs via diffusion or the BOR1 export transporter. (B) Close-up of a cell wall pectin matrix with borate cross-linking two RG-II monomers (only the apiosyl residues are shown for simplicity) and phenylboronate interfering with the cross-linking by competitively binding to only one RG-II monomer. (C) Reaction of the borate anion and the phenylboronate anion with apiosyl residues of RG-II monomers.

The micronutrient boron (B) is one of the essential plant micronutrients (Warington, 1923). B is taken up from the soil in the form of the uncharged boric acid (BA) (Fig. 1A). BA can be taken up either passively (via diffusion) or actively under B-deficient conditions in the soil (Fig. 1A). The range between B deficiency and toxicity is very narrow and therefore proper B homeostasis is required in the plant. B deficiency in plants is often caused by low soil B content, which occurs worldwide, and is reportedly one of the most widespread micronutrient deficiencies (Shorrocks, 1997). Currently, there is an effort in various research laboratories worldwide to understand the effects of B deficiency and toxicity in different plant species including, but not limited to, *Arabidopsis thaliana* (Arabidopsis), *Zea mays* (maize), *Brassica napus* (Brassica), *Oryza sativa* (rice), *Malus domestica* (apple), citrus, *Hordeum vulgare* (barley), and wheat (Noguchi *et al.*, 1997; Takano *et al.*, 2006; Nakagawa *et al.*, 2007; Sutton *et al.*, 2007; Wimmer *et al.*, 2009; Schnurbusch *et al.*, 2010; Tanaka *et al.*, 2011; Miwa *et al.*, 2013; Yang *et al.*, 2013; Abreu *et al.*, 2014; Chatterjee *et al.*, 2014, 2017; Durbak *et al.*, 2014; Hanaoka *et al.*, 2014; Leonard *et al.*, 2014; Pallotta *et al.*, 2014; Liu *et al.*, 2015; Fang *et al.*, 2016; Matthes and Torres-Ruiz, 2016; Eggert and von Wirén, 2017; Sotta *et al.*, 2017; Zhang *et al.*, 2017; Duran *et al.*, 2018; Routray *et al.*, 2018; Shao *et al.*, 2018; Diehn *et al.*, 2019; Gómez-soto *et al.*, 2019).

B deficiency has negative consequences on plant growth, development, and performance. One of the earliest defects detected upon B deficiency is a cessation of growth at the growing tips, or meristems (groups of stem cells, that give rise to all above- and below-ground organs). This growth arrest due to a reduction in cell elongation and cell division (Dell and Huang, 1997; Poza-viejo *et al.*, 2018), in both the root and the shoot, leads to a balanced reduction of the shoot to root biomass. This response is in contrast to other macronutrient deficiencies, for example nitrogen or phosphorus which lead to enhanced root growth, and therefore an increase in the root to shoot biomass (Hermans *et al.*, 2006). While a general growth arrest has also been reported in plants subjected to many other nutrient deficiencies, a negative effect on meristematic tissues is known to occur for only a few others, including calcium, copper, iron, and zinc (Marschner, 2012; Mendoza-Cózatl *et al.*, 2019). Therefore, understanding the molecular mechanisms underlying B deficiency is of interest not only to plant physiologists, but also to developmental biologists.

B has been shown to be functionally involved in the stability of the primary cell wall by cross-linking two of the rhamnogalacturonan-II (RG-II) pectic subunits via their apiosyl residues (Ishii and Matsunaga, 1996; Kobayashi *et al.*, 1996; O’Neill *et al.*, 1996) (Fig. 1B). As cell wall biosynthesis and stability have to be maintained for proper plant development, B has to be continuously delivered to growing tissues from the soil through the root and vascular tissues by directional cell to cell transport. The cross-linking of pectin subunits in the cell wall is so far the best understood, characterized, and, although recently challenged (Lewis, 2019), also the accepted primary role of B (Gonzales-Fontes, 2019; Wimmer *et al.*, 2019). Additional roles for B have been proposed and demonstrated related to metabolism, membrane processes, and phytohormone signaling (discussed below), though it is difficult to separate primary and secondary effects of B deficiency (Blevins and Lukaszewski, 1998; Bolaños *et al.*, 2004; Goldbach and Wimmer, 2007; Wimmer *et al.*, 2009; Voxeur and Fry, 2014). Hypotheses regarding additional roles of B beyond the cell wall are, at least in part, founded on the observation that the amount of B that is localized in cell walls stays relatively constant with varying B concentration of the soil or media, while the amount of cytosolic B follows the B concentration of the soil, suggesting that the cytosolic B pool could be involved in early B deficiency reactions (Goldbach *et al.*, 2000).

In the membrane, roles of B in influencing the function and the localization of plasma membrane (PM) proteins, transport processes across the membrane, and membrane integrity have been demonstrated (Goldbach and Wimmer, 2007; Wimmer *et al.*, 2009; Voxeur and Fry, 2014; Matthes and Torres-Ruiz, 2016). The identification of membrane-associated, B-interacting proteins and the *in vitro* formation of a glycosylinositol phosphorylceramides (GIPC)–B–RG-II complex provide the first molecular explanations for a function of B in the stabilization of membranes/membrane domains and possible wall–membrane attachment sites (Wimmer *et al.*, 2009; Voxeur and Fry, 2014). Although the experimental evidence of the physiological significance of the identified B membrane proteins and the *in vivo* existence of a GIPC–B–RG-II complex is missing, these studies provide pivotal insights into the biochemical basis for the B dependency of plant growth and development.

In recent years, progress in B research has been made in identifying and characterizing mutants with impaired B transport (Noguchi *et al.*, 1997; Takano *et al.*, 2006; Nakagawa *et al.*, 2007; Tanaka *et al.*, 2008; Miwa *et al.*, 2013, 2014; Chatterjee *et al.*, 2014, 2017; Durbak *et al.*, 2014; Hanaoka *et al.*, 2014; Leonard *et al.*, 2014; Liu *et al.*, 2015; Zhang *et al.*, 2017; Shao *et al.*, 2018), understanding the underlying mechanisms of B homeostasis and regulation (Sotta *et al.*, 2017; Yoshinari and Takano, 2017; Yoshinari *et al.*, 2019), development of B imaging techniques (Fukuda *et al.*, 2018; Wang *et al.*, 2018; Zhou *et al.*, 2018; Housh *et al.*, 2020), and studying possible interactions of B with phytohormones (Abreu *et al.*, 2014; Camacho-Cristóbal *et al.*, 2015; Li *et al.*, 2015; Matthes and Torres-Ruiz, 2016; Zhou *et al.*, 2016; Eggert and von Wirén, 2017; Poza-viejo *et al.*, 2018; Gómez-soto *et al.*, 2019). Additionally, B-mediated changes in plant water relations and implications in interactions of B with other abiotic factors, such as drought, is gaining interest in the B research community (Wimmer and Eichert, 2013; Macho-Rivero *et al.*, 2017).

With this review, we evaluate differences and limitations of the published methodologies used to induce B deficiency, in the context of our current understanding of the effects of B deficiency on plant development.

## B homeostasis through concerted action of boric acid facilitators and borate exporter proteins

The B requirement of plants is species specific, yet the range between deficient and toxic soil B concentrations is small (Goldberg, 1997). Therefore, plants regulate B homeostasis in order to maintain optimal B concentrations within their cells. Research in recent years has shed light on the molecular mechanisms that underly the maintenance of B homeostasis (as reviewed in Yoshinari and Takano, 2017). In short, plants control B uptake and transport through rapid regulation of two different classes of B transporters, namely BA channels of the major intrinsic protein (MIP) family and borate transporters of the BOR family (Fig. 1A).

For B to be used by the plant, it has to be (i) in a plant-accessible form, which is either the uncharged BA or the borate anion; and (ii) transported from the soil and through the roots to the shoot. B is taken up by the plant in the form of BA (Fig. 1A), which is the main form of B in solution at physiological pH (5–6). Within a plant cell, the cytosolic pH (7.5) leads to the formation of the borate anion, which readily interacts with apiosyl residues of RG-II in the cell wall (Fig. 1B). Since biological membranes are quite permeable for BA, passive uptake of BA via diffusion prevails when B concentrations are adequate or high (Raven, 1980; Dordas and Brown, 2000). The identification of a necessity for an active B transport mechanism (Dordas *et al.*, 2000) was refined by the characterization of Arabidopsis mutants that require higher B concentrations in the growing medium (Noguchi *et al.*, 1997; Takano *et al.*, 2006). These mutant studies helped establish that under low soil B concentrations, BA is taken up by the plant with the help of BA channels of the MIP family and transported within the plant by borate exporters of the BOR family. The BA channels belong to the nodulin 26-like intrinsic protein (NIP) subfamily of MIPs. NIPs are further divided into the subclasses I, II, and III, of which the subclass II is important for BA uptake (Roberts and Routray, 2017). While Arabidopsis NIP5;1 (*At*NIP5;1) transports B into the root from the soil (Fig. 1A), *At*HIGH BORON REQUIRING1 (*At*BOR1) is involved in the export of B out of the cell (Fig. 1A). Both of these transporter families show polar subcellular localization, which so far has only been shown in Arabidopsis. While *At*BOR1 displays polar localization towards the inner/stele side in various root cell types, the hypocotyl, and cotyledons (Takano *et al.*, 2010; Yoshinari *et al.*, 2019), *At*NIP5;1 shows polar localization towards the soil side in Arabidopsis root epidermal and root cap cells (Takano *et al.*, 2010). Due to their polar localization, the concerted action of *At*BOR1 and *At*NIP5;1 leads to a directional, radial transport of B from the soil into the stele. From there, B is loaded into the xylem and transported through the transpiration stream to the shoot. To regulate B levels within a plant, the abundance of both transporter classes is highly controlled and depends on the cytosolic B concentration. While the abundance of *At*NIP5;1 is primarily regulated via ribosome stalling and subsequent mRNA degradation (Tanaka *et al.*, 2011, 2016), the abundance of *At*BOR1 is regulated via degradation through ubiquitination and translational suppression (Takano *et al.*, 2010; Aibara *et al.*, 2018).

The regulation of B transport occurs on a fast time scale. Endocytotic *At*BOR1 degradation is apparent 30 min after the transfer from low to high B medium and it is suggested that *At*BOR1’s half-life is under an hour (Takano *et al.*, 2005). On the other hand, *AtNIP5;1* mRNA’s half-life is 10–15 min (Tanaka *et al.*, 2011). Using a mathematical modeling approach, it was recently shown that such rapid (also termed ‘swift’ by Sotta *et al.*, 2017) regulation of transporter abundance secures a constant B concentration within root cells. Delaying the transporter regulation swiftness *in silico* leads to (i) oscillations of B concentrations in the simulated cells; (ii) traffic jam-like increases of cellular B concentrations propagating back from the xylem to the soil against the B flow from the soil to the xylem; and (iii) a reduction in total B throughput through the tissue and ultimately reduced xylem loading of B. As the threshold for B toxicity is narrow, increases in B concentrations caused by such oscillations would lead to DNA damage and cell death. This rapid regulation of B transporter abundance therefore is suggested to be one mechanism by which plants avoid high intracellular B levels to limit B-induced damage and to maintain optimal constant B xylem loading rates over time (Sotta *et al.*, 2017). Experimental data substantiating the occurrence of oscillations in B concentrations due to a lack of rapid regulation of B transporters would be a valuable contribution to support this modeling approach.

Other NIP and BOR family members have also been shown to be involved in either B uptake, distribution, or relocation, particularly in Arabidopsis. While *At*NIP6;1 is involved in B transfer from the xylem to the phloem (Tanaka *et al.*, 2008), *At*NIP7;1 was shown to be a B uptake facilitator in developing anthers (Li *et al.*, 2011). *At*BOR1’s closest homolog, namely *At*BOR2, as well as *At*BOR4 were also shown to be involved in B export (Miwa *et al.*, 2013, 2014).

B can be found in various locations in the plant cell including the cell wall, the cytoplasm, and the vacuole (Thellier *et al.*, 1979; Dannel *et al.*, 1998). Additional cellular locations of B, such as the PM, have also been demonstrated (Pollard *et al.*, 1977; Cakmak and Römheld, 1997; Wimmer *et al.*, 2009; Voxeur and Fry, 2014). While the majority of a plant’s B content is reported to be found in the cell wall bound to RG-II (Matoh *et al.*, 1996), it is the cytosolic B content that varies, when B contents in the growth media are altered (Goldbach *et al.*, 2000). Interestingly, the 5'-untranslated region (UTR) of *AtNIP5;1* ‘responds’ to alterations in the cytosolic B content, and was therefore recently used to develop a cytosolic B sensor (Fukuda *et al.*, 2018). To date, the cytosolic B content is suggested to be the sole site of B sensing, but detailed studies of varying B concentrations in other cellular locations are lacking. Water-soluble B (cytosolic B) is usually measured when reporting B contents/concentrations in plants (see Supplementary Table S1 at *JXB* online), and reports that quantify B–RG-II dimers together with B content/concentrations are scarce (Supplementary Table S1), but are necessary to elucidate a more complete picture of B’s cellular function(s) in plants.

## How to study B deficiency

B is ubiquitously present, making it difficult to experimentally induce B deficiency in a controlled manner on a plant level. Typically there are three approaches that are used by the research community, namely (i) making use of particular mutants in either the BA import facilitators or the borate export proteins (as outlined in the first section); (ii) using chemicals, such as boronic acids [e.g. phenylboronic acid (PBA), Fig. 1] to mimic B deficiency; or (iii) transfer assays from B-containing medium onto medium with reduced B (either solid medium or hydroponic cultures). The mutant approach has successfully been used in various plant species, including Arabidopsis (Noguchi *et al.*, 1997; Takano *et al.*, 2006), maize (Chatterjee *et al.*, 2014, 2017; Durbak *et al.*, 2014; Leonard *et al.*, 2014), Brassica (Zhang *et al.*, 2017), and rice (Nakagawa *et al.*, 2007; Hanaoka *et al.*, 2014; Liu *et al.*, 2015). Due to reduced uptake or distribution of B, these mutants typically have a reduced cytosolic B content and, for some of them, reduced B–RG-II cross-linking has been reported as well (Supplementary Table S1). Therefore, the mutant approach can be used to study the effects of the reduction of cytosolic B content as well as the effects of the reduction in B–RG-II cross-linking. An exception to this is the *Atbor2* mutant, which was shown to not influence cytosolic B content, but rather solely interferes with B–RG-II cross-linking in the cell wall (Miwa *et al.*, 2013) (Supplementary Table S1). Unfortunately, analysis of B–RG-II complexes in addition to cytosolic B content/concentration measurements has been reported in only a few studies (Noguchi *et al.*, 2000, 2003; Miwa *et al.*, 2013; Durbak *et al.*, 2014) (Supplementary Table S1), yet would provide important data for elucidating potential cell wall-independent roles of B.

Limitations of using a mutant approach are that (i) deciphering cause versus consequence of B deficiency is difficult, since typically the end-product is examined, for example, the formation of a particular structure or organ; and (ii) phenotypes can be highly variable, especially when studying crops, due to non-standardized or non-controllable conditions in the field (Fig. 3, note asterisks in B, C, and E). In order to gain deeper insights into the primary roles of B on a cellular level, different approaches are needed. Recently, it has been suggested that chemical approaches, which interfere with RG-II dimerization, might provide an avenue for that. Suitable chemicals are, for example, boronic acids (Bassil *et al.*, 2004; Matthes and Torres-Ruiz, 2017) or inhibitors of fucosylation of cell wall components (Dumont *et al.*, 2015), since they can interfere with specific biochemical processes in a transient way and therefore allow a precise induction of B deficiency in a timely manner. Boronic acids, such as PBA, interfere with BA’s capability to bind and cross-link *cis*-diol groups, specifically RG-II in the cell wall (Bassil *et al.*, 2004) (Fig. 1B, C). They interfere in this process by only binding two *cis*-diols and, therefore, inhibiting the cross-linking of two RG-II molecules in the cell wall (Fig. 1B, C). Boronic acids have so far been used to induce B deficiency in tobacco (Bassil *et al.*, 2004), petunia (Bassil *et al.*, 2004), Arabidopsis (Matthes and Torres-Ruiz, 2016; Duran *et al.*, 2018), and apple (Fang *et al.*, 2016). The chemical approach will lead to an increase in cytosolic B content, rather than a decrease, as boronic acids will still deliver B into the cell (Duran *et al.*, 2018) and B deficiency-like symptoms are assumed to be solely due to an interference with cell wall cross-linking (Bassil *et al.*, 2004; Matthes and Torres-Ruiz, 2016). However, direct effects of boronic acids on transcription or other processes/molecules that offer *cis*-diols have not been excluded. Additionally, boronic acids have been introduced as small-molecule auxin inhibitors that target YUCCA auxin biosynthesis enzymes (Kakei *et al.*, 2015), which might provide evidence of a direct interaction between B and auxin function which is disrupted by the boronic acid. Alternatively, boronic acids might not be specific to inducing B deficiency which should be a target of future research. By combining a mutant and a chemical approach, a dissection of the effects of cytosolic B deficiency versus disruption of cell wall stability/integrity might be possible.

In the third approach, transfer assays make use of solution cultures, where plants are initially grown with adequate levels of B and afterwards transferred to conditions with an insufficient amount of B. The plant’s response to B withdrawal is measured. Solution cultures are performed in either solid or liquid medium. In contrast to conventional solution cultures, B-free medium is used to which specific amounts of B are subsequently added. In order to obtain B-free medium, the use of glassware is avoided in the preparation of buffers, and B-chelating resins such as Amberlite® IRA743 are used (Bell *et al.*, 2002). The use of Amberlite® IRA743 additionally allows buffering of B in the nutrient solution (Asad *et al.*, 1997) and is reported to maintain B concentrations over multiple days (Huang *et al.*, 1999), which is not the case in conventional solution cultures, where nutrient concentrations may vary due to infrequent renewal of the culture medium. The effectiveness of Amberlite® IRA743 in maintaining B concentrations depends on various factors and therefore should be determined for each experimental set-up. Like the chemical approach, the analysis of short periods of B withdrawal in B-buffered solution cultures using Amberlite® IRA743 allows the study of primary or early secondary effects upon B deficiency. In combination with a mutant approach, B-buffered solution cultures are therefore a powerful technique for controlled investigations on B nutrition.

In addition to the aforementioned approaches to induce B deficiency, there are several breeding approaches and strategies that are being applied or tested to enhance B availability and uptake in plants, which add to the techniques to study B nutrition and B deficiency in plants. Among those techniques are methods to modify root traits, grafting of suitable rootstocks, application of biostimulators (materials other than fertilizers that influence plant metabolic processes) or nano-fertilizers, and inoculation with mycorrhizal fungi or plant growth-promoting rhizobacteria, as reviewed in Shireen *et al.* (2018). In addition, transgenic approaches using B transport proteins appear promising for enhancing crop productivity under both B deficiency and toxicity conditions (Miwa *et al.*, 2006, 2007; Kato *et al.*, 2009; Takada *et al.*, 2014; Uraguchi *et al.*, 2014; Wakuta *et al.*, 2016).

## Effects of B deficiency on plant development

**Fig. 2.**
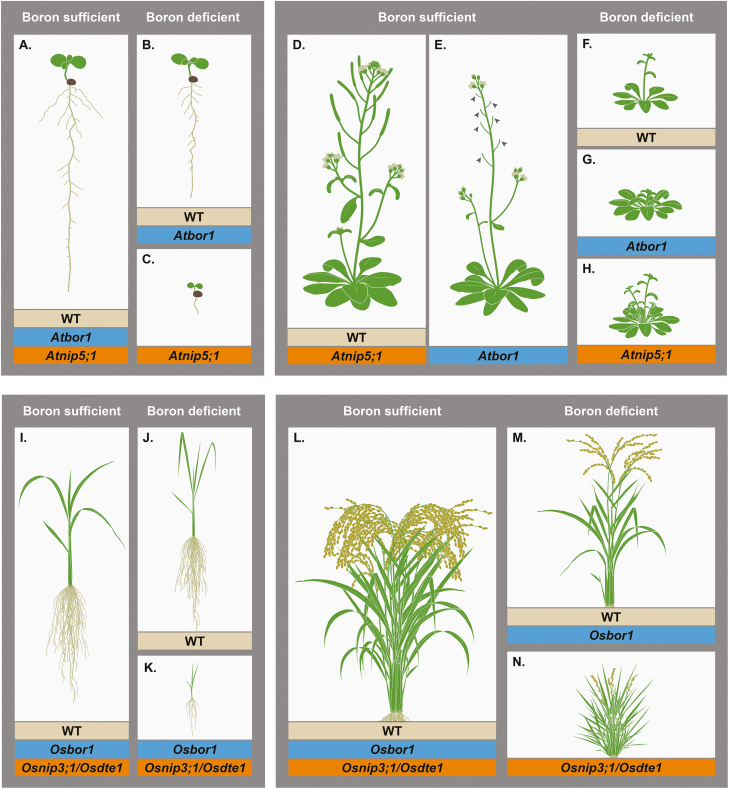
Phenotypes of the Arabidopsis and rice *bor1* and *nip5;1* transporter mutants when grown under B-sufficient and B-deficient conditions. Root phenotypes, when grown under B-sufficient conditions of (A) wild-type Arabidopsis (WT), *Atnip5;1*, and *Atbor1*. Root phenotypes, when grown under B-deficient conditions of (B) WT, *Atbor1* and (C) *Atnip5;1*. Reproductive phenotypes, when grown under B-sufficient conditions of (D) WT, *Atnip5;1* and (E) *Atbor1* (arrowheads depict reduced seed set due to female sterility). Reproductive phenotypes, when grown under B-deficient conditions of (F) WT, (G) *Atbor1*, and (H) *Atnip5;1*. (I) Vegetative phenotypes, when grown under B-sufficient conditions of WT rice, *Osbor1*, and *Osdte1*/*Osnip3;1* mutants. Vegetative phenotypes, when grown under B-deficient conditions of (J) WT rice, and (K) *Osbor1* and *Osdte/Osnip3;1*. (L) Reproductive phenotypes, when grown under B-sufficient conditions of WT rice, *Osbor1*, and *Osdte1/Osnip3;1*. Reproductive phenotypes, when grown under B-deficient conditions of (M) WT rice, *Osbor1*, and (N) *Osdte1/Osnip3;1*.

**Fig. 3.**
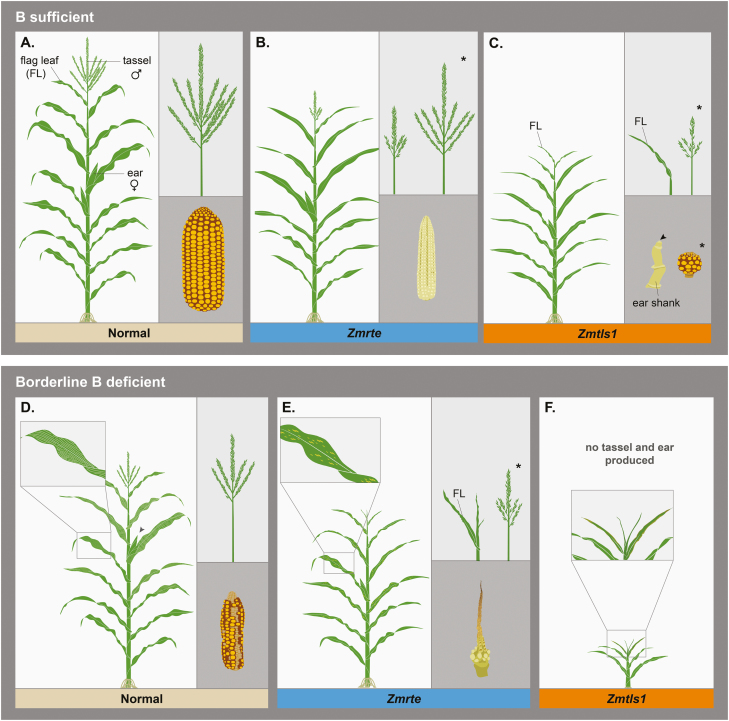
Phenotypes of normal maize plants, and *Zmrte* (co-ortholog of *Atbor1*) and *Zmtls1* (co-ortholog of *Atnip5;1*) transporter mutants, when grown under B-sufficient and B-deficient conditions. Vegetative and reproductive phenotypes, when grown under B-sufficient conditions of (A) normal maize plants, (B) *Zmrte*, and (C) *Zmtls1* (note that the arrowhead points to a missing ear; depicted is only the ear shank, a structure typically subtending an ear). Vegetative and reproductive phenotypes, when grown under borderline B-deficient conditions of (D) normal maize plants, (E) *Zmrte*, and (F) *Zmtls1*. The asterisk indicates phenotypic variability. FL=flag leaf; male and female signs indicate a male and female inflorescence, respectively.

The study of B deficiency shows that B is necessary for proper plant development, as B deficiency leads to root, leaf, flower, and meristem defects in various species, including Arabidopsis, maize, and rice (Figs 2, 3). Reports on the absolute internal and external B requirements for a given plant species (Hu *et al.*, 1996; Bell *et al.*, 2002; Lordkaew *et al.*, 2011; Eggert and von Wirén, 2017) point out several common findings: (i) B requirements for growing organs exceed the requirement of mature organs; (ii) B requirements in reproductive plant parts are higher than in vegetative plant parts; and (iii) internal B requirements appear to be highly species specific, which seems to be correlated with the composition of the primary cell wall (Hu *et al.*, 1996). The higher B requirement of reproductive tissues might explain why reproductive growth is particularly sensitive to B limitation and the fact that seed yield reductions have been reported without any other symptoms during vegetative growth (Dell and Huang, 1997; Lordkaew *et al.*, 2011).

Interestingly, mutant phenotypes of characterized B transporters only partially match up with the phenotypes observed when normal plants are grown under B deficiency (Figs 2, 3). For example, wild-type (WT) Arabidopsis plants grown without any B (hydroponic culture: no added B) retain apical dominance, and exhibit severe defects in rosette leaf expansion (Noguchi *et al.*, 1997) (Fig. 2F). When grown under B-deficient conditions (solid medium: 0.1 µM B), WT Arabidopsis plants show only a moderate reduction in primary root length and still develop lateral roots (Takano *et al.*, 2006) (Fig. 2B). In contrast, *Atbor1* mutants display severe repression of apical dominance and a moderate reduction in the expansion of the rosette leaves, when grown under B-deficient conditions (hydroponic culture: 3 µM B) (Fig. 2G). Root growth is only altered once leaf growth becomes severely affected (Noguchi *et al.*, 1997). In addition, *Atbor1* fails to set seed due to female sterility (Noguchi *et al.*, 1997) (Fig. 2E; note arrowheads that point to sterile flowers) when grown under B-sufficient conditions (hydroponic culture: 30 µM B). On the other hand, *Atnip5;1* mutants display severe root defects when grown under B-deficient conditions (hydroponic culture: 3 µM B) (Fig. 2C) including a cessation of primary root growth, absence of lateral roots, an increase in root hair density, no further expansion of the rosette leaves, and a striking inhibition of the development of flowers and siliques (Takano *et al.*, 2006) (Fig. 2C, H). The ‘nude’ root phenotype (no lateral roots) and a reduction in expansion of the rosette leaves persist with the addition of moderate B levels. With adequate B levels (hydroponic culture: 30 µM B), the *Atnip5;1* mutant does not display any developmental phenotypes and grows similarly to WT Arabidopsis accessions (Takano *et al.*, 2006) (Fig. 2D). In conclusion, *Atbor1* and *Atnip5* mutants have different phenotypes from each other that are more severe than those of WT Arabidopsis plants grown in B-deficient conditions (Fig. 2).

Young rice seedlings do not show any plant height or root lengths defects when grown under B deficiency (hydroponics: 0.03 µM B) for <4 weeks (Uraguchi and Fujiwara, 2011). Prolonged growth under B deficiency (hydroponics: 0.18 µM B or 0.03 µM B) leads to a reduction in root length, plant height, and grain yield, due to low fertility and reduction in the number of panicles (inflorescence of rice) and spikelets (flower-harboring structure in grasses) (Uraguchi and Fujiwara, 2011) (Fig. 2J–M). T-DNA insertion lines in the rice ortholog of *AtBOR1*, namely *Osbor1*, show severe reductions in both shoot and root growth in comparison with WT rice plants when grown for 4 weeks under B-deficient conditions (hydroponics: 0 µM B) (Nakagawa *et al.*, 2007) (Fig. 2K). *OsNIP3;1/DWARF AND TILLER ENHANCING1* (*DTE1*) is the rice homolog of *AtNIP5;1* (Hanaoka *et al.*, 2014; Liu *et al.*, 2015). When grown under B-sufficient conditions (hydroponics: 18 µM B), *Osnip3;1/Osdte1* RNAi knockdown lines do not differ morphologically from WT rice, whereas when grown for 3 weeks under B-deficient conditions (hydroponics: 0 µM B), *Osnip3;1/Osdte1* RNAi lines showed severe shoot growth reduction in contrast to WT rice (Fig. 2K) (Hanaoka *et al.*, 2014). Under low B field conditions, *Osnip3;1*/*Osdte1* mutants show enhanced tillering, a reduction in plant height, reduced seed set, and reduced pollen viability (Fig. 2N), while B-sufficient field conditions do not lead to any obvious growth defects of *Osnip3;1/Osdte1* in comparison with WT rice (Liu *et al.*, 2015).

In maize, B deficiency negatively influences yield, yet, in comparison with other cereals, maize has been reported to have a relatively low B requirement for maximum yield (Martens and Westermann, 1991). When grown under B deficiency (e.g. in sand without additional B), no vegetative phenotypes are seen in normal maize plants, although B concentrations can already be significantly reduced within the plant (Lordkaew *et al.*, 2011). When entering the reproductive stage, the upper leaves exhibit translucent streaks (Fig. 3D) and plants produce multiple ears (female inflorescence in maize), that might harbor a tassel-like (male inflorescence in maize) structure, with few short silks (stigmas of maize ears). In addition, tassels are visibly smaller (Fig. 3D), with small, shriveled anthers, that can in extreme cases be devoid of pollen (Lordkaew *et al.*, 2011). Similar phenotypes are seen in maize mutants co-orthologous to *Atbor1* and *Atnip5;1*, namely *Zmrotten ear* (*Zmrte*) (Chatterjee et al., 2014, 2017) and *Zmtassel-less1* (*Zmtls1*) (Durbak *et al.*, 2014; Leonard *et al.*, 2014; Matthes *et al.*, 2018). *Zmrte* shows defects in both male and female reproductive development (Fig. 3B, E). In severe cases, tassels are shorter and less branched with no spikelets (Fig. 3E). Severe ear defects include being shorter, shrunken, withered, and not producing mature spikelets (Chatterjee *et al.*, 2014). *Zmrte* phenotypes are variable depending on the genetic background and the B content in the soil, so that both tassel and ear can appear rather normal (note the asterisk in Fig. 3B), but are always sterile irrespective of B concentration in the soil (Fig. 3B, E). *Zmrte* also displays loss of apical dominance and shows occasional necrotic lesions on leaves, and shorter, discolored, or wrinkly leaves (Chatterjee *et al.*, 2014) (Fig. 3E). The vegetative leaf phenotypes are different in morphology from the reported transparent streaks on leaves, when normal maize is grown in sand without added B (Lordkaew *et al.*, 2011) (compare Fig. 3D and E). The defects seen in *Zmrte* mutants, grown under B-deficient conditions, are enhanced when the action of its duplicated gene *Zmrte2* is also impaired (Chatterjee *et al.*, 2017). *Zmrte;rte2* double mutants produce only few leaves and die after a few weeks when grown in B-deficient soil (Chatterjee *et al.*, 2017). The severe vegetative defect is reminiscent of *Zmtls1* mutants, when grown under borderline B-deficient conditions (Durbak *et al.*, 2014) (Fig. 3F), where *Zmtls1* mutants develop only 4–8 narrow, stiff leaves, and die at the seedling stage (Durbak *et al.*, 2014; Matthes *et al.*, 2018). A similarly severe vegetative phenotype can be obtained by growing normal maize in a nutrient solution without added B (Eltinge, 1936). In contrast to reported *Zmrte* or *Zmtls1* phenotypes, the first visible defect in normal maize grown in nutrient solution without added B is a yellow discoloration between the veins of older leaves and an unrolling of the youngest visible leaf (Eltinge, 1936). In B-rich soils, *Zmtls1* displays variable reproductive defects, including not developing a tassel or producing a highly reduced tassel (Fig. 3C; note asterisk). In addition, the ear is either completely absent or short and ball-shaped (Fig. 3C). While both *Zmtls1* and *Zmrte* can develop multiple ears on the same node like normal maize grown in sand without added B, neither mutant has been reported to develop a tassel instead of an ear.

The study of developmental and growth defects in different species under B deficiency shows interesting differences in phenotypes and in regulation of B transporters. Such differences might indicate that regulatory responses upon B deficiency are species specific, therefore necessitating future research efforts for B regulation in various species, from both a physiological and a developmental point of view. Examples for species-specific differences are: (i) *Zmrte* shows defects in both male and female reproductive development, which is different from the *Atbor1* mutant; (ii) the root phenotype of the *Osbor1* T-DNA insertion lines is in contrast to both *Atbor1* and *Zmrte* mutants, which do not show strong root phenotypes; (iii) *Os*BOR1 is involved in xylem loading, but, unlike *At*BOR1, is also functionally important for B uptake into roots (Nakagawa *et al.*, 2007); and (iv) *Os*BOR1’s role in xylem loading is independent of the B conditions, whereas *At*BOR1 is reported to be less important under B-sufficient conditions (Nakagawa *et al.*, 2007). It has to be noted, though, that *At*BOR1’s role under B-sufficient conditions might have been overlooked, as B concentrations in the *Atbor1* mutant are severely reduced specifically in the upper portion of the inflorescence even under B-sufficient conditions (hydroponics: 30 µM B) (Noguchi *et al.*, 1997). (v) While WT rice plants typically accumulate B in the leaf sheath in comparison with the leaf blade, the *Osnip3;1* RNAi knockdown mutant accumulates more B in the leaf blades compared with the leaf sheath (Hanaoka *et al.*, 2014). Indeed, it was recently shown that *Os*NIP3;1 is not involved in B uptake or translocation, but rather in preferentially delivering B to developing tissues (Shao *et al.*, 2018).

It was recently shown by using a chemical approach with PBA that B deficiency leads to very specific developmental defects when applied during Arabidopsis embryogenesis. The application of PBA was shown to specifically lead to the disruption of the asymmetric cell division of the hypophysis (the precursor cell for the root apical meristem; RAM) (Matthes and Torres-Ruiz, 2016). Consequently, RAM formation was blocked, leading to phenocopies of the *At*AUXIN RESPONSE FACTOR 5/MONOPTEROS (*mp*) mutants. *Atmp* mutants fail to perform the correct cell division of the hypophysis and give rise to seedlings with no primary root (Berleth and Jürgens, 1993). The primary defect upon PBA treatment in Arabidopsis embryos was hypothesized to be a transient delocalization of the polarly localized auxin efflux carrier *At*PINFORMED1 (PIN1), leading to a disruption of the directional flow of the phytohormone auxin. The very specific induction of *Atmp* phenocopies upon inducing B deficiency in Arabidopsis embryos suggests an interaction of B with auxin and adds data to support the long-standing hypothesis of additional roles of B beyond its cell wall function (Brown *et al.*, 2002).

## Interactions of B and hormones

Potential interactions between B and in particular the hormones auxin, cytokinin (CK), ethylene, and abscisic acid (ABA) have been hypothesized (Blevins and Lukaszewski, 1998; Wang *et al.*, 2006; Abreu *et al.*, 2014; Li *et al.*, 2015; Matthes and Torres-Ruiz, 2016; Macho-Rivero *et al.*, 2017; Poza-viejo *et al.*, 2018; Gómez-soto *et al.*, 2019). While direct evidence for such interactions is missing, they would lead to the elucidation of additional roles of B beyond the cell wall and help determine if hormonal defects are a secondary effect of an altered cell wall and consequently an altered morphology, or if B interacts with hormones directly. Specifically, there is a long-standing interest in studying interactions of B with auxin, as B deprivation was shown to cause alterations of levels of the main active auxin indole 3-acetic acid (IAA) as reviewed in Blevins and Lukaszewski (1998). These alterations are hypothesized to be due to (i) enhancement of the activity of IAA oxidase, an enzyme involved in the catabolism of IAA (Jarvis *et al.*, 1983); or (ii) the involvement of B in auxin transport (Li *et al.*, 2001; Wang *et al.*, 2006; Li *et al.*, 2015; Matthes and Torres-Ruiz, 2016). Interestingly, numerous studies in various plant species have either directly or indirectly shown alterations of auxin levels upon B deficiency, but with different results (Blevins and Lukaszewski, 1998; Martín-Rejano *et al*., 2011; Li *et al.*, 2015; Matthes and Torres-Ruiz, 2016; Zhou *et al.*, 2016; Eggert and von Wirén, 2017), hindering a general reasoning of the relationship between B deficiency and hormones, such as auxin. Reasons for such potential discrepancies could be attributed to different levels of B deficiencies, different organs being analyzed, and different methods of inducing B deficiency. This indicates not only the necessity for standardized methodologies, but also the necessity of reporting B deficiency symptoms/effects in defined cellular locations, tissues, and developmental stages in order to make studies comparable and reproducible.

**Fig. 4.**
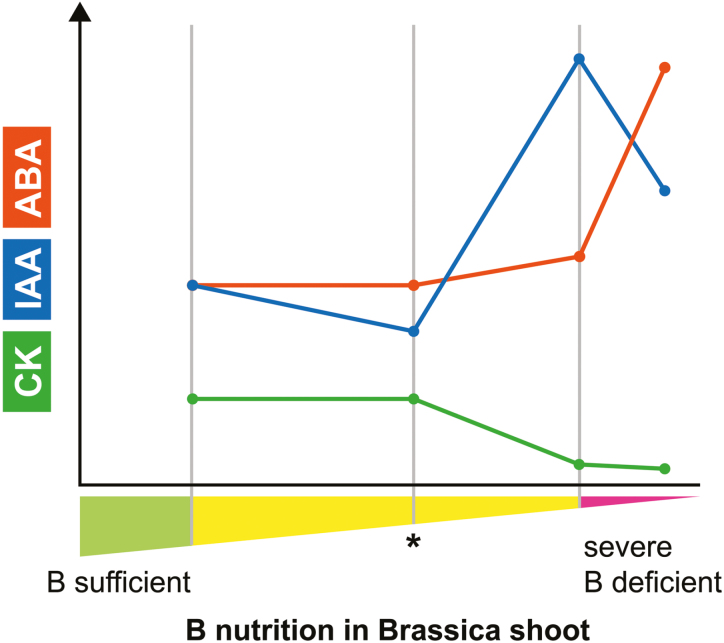
Trends of IAA, ABA, and CK levels with increasing B deficiency in *Brassica napus* plants. Modified from Eggert and von Wirén (2017) with permission.

A recent study in Brassica seedlings (Eggert and von Wirén, 2017), which are highly sensitive to B deficiency (Zhang *et al.*, 2014), highlights this importance. Eggert and von Wirén (2017) took a physiological approach, where they simultaneously measured active phytohormone species, including their precursors and catabolites, in association with the shoot B nutritional status to detect B-dependent growth responses. This study revealed that until the presence of B deficiency symptoms are manifest (Fig. 4; note the asterisk), IAA levels slightly decrease, but afterwards the concentration of all tested auxin species increased with progressing B deficiency (Eggert and von Wirén, 2017) (Fig. 4). When crossing a certain threshold of B deficiency, however, IAA levels significantly dropped, although they were still higher than under B-sufficient conditions (Fig. 4). The authors used these opposing behaviors of IAA levels depending on the B nutritional status of a plant to explain contradictions between their study and the study by Zhou *et al.* (2016), which reported reduction in shoot IAA concentrations in Brassica under low B supply.

Contradictions about the level of IAA in B-deficient conditions have also been reported in Arabidopsis (Li *et al.*, 2015; Matthes and Torres-Ruiz, 2016). For example, by using the synthetic auxin signaling marker DR5:GFP (green fluorescent protein) in Arabidopsis embryos, a reduction of GFP in the RAM was observed upon PBA application to siliques (Matthes and Torres-Ruiz, 2016), indicating reduced auxin levels in the embryonic RAM upon B deficiency. A different study suggested an overaccumulation of auxin in the Arabidopsis root stele, as detected by the absence of the DII-Venus reporter line, which is degraded when auxin is present (Li *et al.*, 2015). In this study, seedlings were grown on agar plates with different B concentrations (normal, 30 µM B; and low, 0.1 µM B) and analyzed 10 d after sowing. While likely reasons for the opposing changes in IAA levels between the two studies are (i) differences in the methods of inducing B deficiency and (ii) differences in the tissue analyzed, it is possible that the observed differences between the two studies lies in the difference of B nutritional status within the plants, as suggested by Eggert and von Wirén (2017). Both Arabidopsis studies nevertheless point to effects of B deficiency on polar auxin transport and the PIN proteins, particularly *At*PIN1. Although the effect of B deficiency on *At*PIN1 leads to different outcomes in the different tissues/cells (overaccumulation of auxin versus a decrease of auxin levels), these findings open up the possibility that *At*PIN1 protein abundance/localization might be one of the first targets upon B deficiency. Although direct evidence for this speculation is missing, it is supported by the observation that other nutrient deficiencies, such as of manganese or calcium, do not affect the abundance of *At*PIN1 (Li *et al.*, 2015). Another hypothesis is that the effect of *At*PIN1 abundance and localization upon B deficiency might be secondary due to the modulation of other hormones such as CK, for example (Li *et al.*, 2015). Elucidating cause versus consequence between B deficiency and IAA metabolism/transport remains a subject for future research.

Although interactions between CK and B status are not as widely explored as those with B and auxin, there are a number of reports demonstrating an involvement of CK in the regulation of the B stress response in plants. In *Pisum sativum* (pea) shoots, a reduction of CK was reported, when grown under B deficiency (hydroponics: 0 µM added B) (Li *et al.*, 2001). In Arabidopsis, it was observed that a CK receptor gene, *CYTOKININ RESPONSE 1* (*CRE1*)/*WOODEN LEG* (*WOL*)/*ARABIDOPSIS HISTIDINE KINASE4* (*AHK4*), is down-regulated upon B deficiency and this down-regulation is correlated with a phenotype of abnormal protoxylem differentiation, proposed to be due to failure in the transition from cell division to cell differentiation (Abreu *et al.*, 2014). A dependence of cytokinin receptor expression on the B nutritional status was also shown in navel oranges (*Citrus sinensis* Osb.) (Yang *et al.*, 2013). Interestingly, the cytokinin receptor gene *WOL* was up-regulated at all time points of increasing B deficiency when compared with the control conditions. The analytical approach in Brassica, that was mentioned earlier (Eggert and von Wirén, 2017), revealed that the active CK forms isopentenyladenine and *cis*-zeatin, as well as the precursors and transport forms, isopentenyladenine riboside and *cis*-zeatin riboside, significantly decreased with increasing B deficiency in Brassica shoots (Eggert and von Wirén, 2017) (Fig. 4). It will be interesting to determine in the future whether the expression of the receptor genes depends on CK levels, which would allow the hypothesis that the transcriptional response of the CK receptors might be a secondary response of the down-regulation of CK levels upon B deficiency. In support of this hypothesis, recent work by Poza-viejo *et al.* (2018) showed increases in CK signaling components as early events after Arabidopsis seedlings had been transferred to B deficiency conditions (solid medium; 0.1 µM B).

An interaction of B with various steps in phytohormone pathways is also highlighted by results from (i) transcriptome studies in B deficiency mutants (–/+ B) versus normal plants or normal plants growing under B-sufficient conditions and then transferred onto B-deficient conditions; and (ii) from the similarity of B-deficient and hormone-deficient phenotypes. For example, transcriptome studies in maize seedlings of the *Zmtls1* mutant revealed that MADS box transcription factors as well as genes involved in the auxin pathway are enriched in untreated *Zmtls1* plants in comparison with untreated normal plants or B-treated *Zmtls1* mutants (Leonard *et al.*, 2014). In addition, some weaker alleles of *Zmtls1* show enhanced tillering, resembling auxin biosynthesis mutants in maize (Durbak *et al.*, 2014).

A novel approach to understanding the interactions between B and various phytohormones comes from a study that analyzed the hormone-responsive motifs in the *AtNIP5;1* promoter (*pAtNIP5;1*) (Gómez-soto *et al.*, 2019). *pAtNIP5;1* was shown to harbor multiple *cis*-related regulatory elements associated with ABA, auxins, CK, salicylic acid, ethylene, gibberellins, and brassinosteroids, suggesting hormonal control of *AtNIP5;1* expression. Indeed, the authors showed induction of *AtNIP5;1* expression with ABA and the ethylene precursor 1-aminocyclopropane-1-carboxylic acid in different parts of the Arabidopsis root especially under B deficiency (Gómez-soto *et al.*, 2019). On the contrary, auxin as well as blocking auxin export most prominently induced *AtNIP5;1* when transferred from B-deficient (solid medium: no added B) to control conditions. While the addition of CK did not alter *AtNIP5;1* expression, inhibiting CRE1 led to an induction of *AtNIP5;1* expression when seedlings were transferred to B-deficient conditions (solid medium: no added B) (Gómez-soto *et al.*, 2019). The findings in this study are interesting, because the induction of *AtNIP5;1* expression by ABA correlated with an increased B uptake (while the induction of *AtNIP5;1* expression by ethylene did not). The correlation between ABA-induced *AtNIP5;1* expression and increased B uptake was speculated to function as an early B stress response. Indications of B interactions with ABA initially came from B toxicity studies, where it was shown that upon B toxicity, ABA content in the shoot increased, which was correlated with an increased expression of the ABA biosynthesis gene *AtNINE-CIS-EPOXYCAROTENOID DIOXYGENASE 3*, in the root (Macho-Rivero *et al.*, 2017). It was also recently shown that ABA and its glycosylester both increased upon severe B deficiency in Brassica (Eggert and von Wirén, 2017) (Fig. 4).

In addition, recent studies indicate the participation of a signaling pathway involving auxin and ethylene in the response of root growth to B deficiency (Camacho-Cristóbal *et al.*, 2015) though it remains challenging to unequivocally distinguish between primary and secondary effects. Approaches to separating out the complexity of primary and secondary effects of B deficiency include the analysis of hormone mutants under short-term B deprivation in solution culture or using a chemical approach by adding hormones to the solution culture and analyzing their effects on plant development under B deficiency (Camacho-Cristóbal *et al.*, 2015; Li *et al.*, 2015; Poza-viejo *et al.*, 2018; Gómez-soto *et al.*, 2019).

## Conclusion and future directions

Significant progress has been made over the last few years particularly in understanding how B homeostasis is achieved/maintained and how/why B deficiency and toxicity affect plant development. Nevertheless, the understanding of the regulatory mechanisms underlying B deficiency is still incomplete. This is in part due to non-standardized methodologies for inducing B deficiency or toxicity, yet standardized methodologies are urgently needed in order to make experiments between groups comparable and repeatable.

To date, B transporters and channels have been associated with regulating B homeostasis in plants, yet their mutant phenotypes do not completely match up with phenotypes observed when normal plants are grown under B deficiency, as outlined above. These observations have been attributed to cell-specific transporter functions and might further indicate that there are additional genes responsible for regulating the B homeostasis in plants. The characterization of mutants that can tolerate low B concentrations in the soil, yet are not B transporters, such as the *low boron tolerance* (*lbt*) mutant in Arabidopsis (Huai *et al.*, 2018) or mutants that interfere with the RG-II cross-linking, such as *murus1* (O’Neill *et al.*, 2001) or the GDP-sugar transporter mutant *gonst3/gglt1* (Sechet *et al.*, 2018), point to a more complex B regulation in plants than currently understood. These findings necessitate future research to elucidate additional molecular players, which will close gaps in our current understanding of how B functions within a plant.

One of the least understood factors in B research is how B is sensed in a cell. While studies with *At*NIP5;1 and specifically its B-dependent regulation suggest that B sensing happens through detection of the intercellular B concentrations, there are several observations that challenge a sensing mechanism that is solely based upon the detection of the intercellular B concentrations. Studies on BOR1–GFP Arabidopsis plants using PBA and BA in the same medium show a strong localization of BOR1–GFP at the PM (Matthes and Torres-Ruiz, 2016), which allows the speculation that B is at least in part sensed at the cell wall, since cytosolic BA levels are still high yet BOR1–GFP is retained at the PM, instead of being degraded. It cannot be excluded that cells employ multiple B-sensing mechanisms, the detection and molecular basis of which should be the focus of future research in the field.

B sensing and deciphering the additional roles of B is still challenging, because subcellular visualization of B or B–RG-II within plants remains demanding, mainly due to the low abundance of B that falls below typical detection levels. Instrumental in tackling these challenges will be the recent development of a B sensor in Arabidopsis, based on the *AtNIP5;1* 5'-UTR that responds to cytosolic B concentrations, which allows visualization of cytosolic B content at a cellular resolution (Fukuda *et al.*, 2018). Additional recent advances in B imaging include the development of an RG-II antibody (Zhou *et al.*, 2018), the employment of quantitative neutron capture radiography on developing maize meristems (Wang *et al.*, 2018), and the development of a B radiotracer based on a PBA derivative (Housh *et al.*, 2020)

Soon the discovery of the essentiality of B will celebrate its 100th anniversary (Warington, 1923), and we still lack a detailed understanding of how B shapes morphological processes. In particular, the dissection of the complexity between primary and secondary responses of B withdrawal on plant development needs in-depth characterization. Treatments that exclusively disrupt meristem growth or B–RG-II formation should be tested and the effects on subsequent hormonal regulation should be contrasted with B deficiency effects. Although B is still the least understood micronutrient, its importance in agriculture continues to advance research into this perplexing element.

## Supplementary data

Supplementary data are available at *JXB* online.

Table S1. B concentration and B-RGII cross-linking in various B transporter mutants under B-deficient and B-sufficient conditions

eraa042_suppl_Supplementary_Table_S1

## Abbreviations:

ABA: abscisic acid
AHK4: ARABIDOPSIS HISTIDINE KINASE4
B: boron
BA: boric acid
BOR1: high boron requiring1
CK: cytokinin
CRE1: CYTOKININ RESPONSE1
DTE: dwarf and tiller enhancing1
GFP: green fluorescent protein
GIPC: glycosylinositol phophorylceramide
IAA: indole 3-acetic acid
MIP: major intrinsic protein
MP: MONOPTEROS
NIP: nodulin 26-like intrinsic protein
PBA: phenylboronic acid
PIN1: PINFORMED1
PM: plasma membrane
RAM: root apical meristem
RG-II: rhamnogalacturonan-II
rte: *rottenear*
SAM: shoot apical meristem
tls1: *tassel-less1*
UTR: untranslated region
WOL: WOODEN LEG
WT: wild type
