# Supplementary material for: From element to development: the power of the essential micronutrient boron to shape morphological processes in plants
Source: J Exp Bot. 2020 Jan 27;71(5):1681–93. doi: 10.1093/jxb/eraa042 (PMC7067301; doi:10.1093/jxb/eraa042)
Supplement: eraa042_suppl_Supplementary_Table_S1 [file eraa042_suppl_supplementary_table_s1.pdf]

Mutant	Tissue	B concentration in relation to WT plant	Relative Proportion of RG-II-B Dimer in comparison to WT	B conditions growing media	Growing conditions	Reference
Atbor1-1	first to fourth rosette leaves	66%	na	30 µm B	Hydroponics	Noguchi et al., 1997
Atbor1-1	upper portion of inflorescence (3 cm from top)	18%	na	30 µm B	Hydroponics	Noguchi et al., 1997
Atbor1-1	leaves and inflorescences	< 50%	na	12 µm B	Hydroponics	Noguchi et al., 1997
Atbor1-1	cell sap shoots	5%	na	3 µm B	Hydroponics	Noguchi et al., 2000
Atbor1-1	cell sap shoots	65%	na	30 µm B	Hydroponics	Noguchi et al., 2000
Atbor1-1	cell sap shoots	100%	na	100 µm B	Hydroponics	Noguchi et al., 2000
Atbor1-1	cell sap roots	67%	na	3 µm B	Hydroponics	Noguchi et al., 2000
Atbor1-1	cell sap roots	100%	na	30 µm B	Hydroponics	Noguchi et al., 2000
Atbor1-1	cell sap roots	80%	na	100 µm B	Hydroponics	Noguchi et al., 2000
Atbor1-1	water insoluble residues shoots	58%	na	3 µm B	Hydroponics	Noguchi et al., 2000
Atbor1-1	water insoluble residues shoots	92%	na	30 µm B	Hydroponics	Noguchi et al., 2000
Atbor1-1	water insoluble residues shoots	102%	na	100 µm B	Hydroponics	Noguchi et al., 2000
Atbor1-1	water insoluble residues root	96%	na	3 µm B	Hydroponics	Noguchi et al., 2000
Atbor1-1	water insoluble residues root	108%	na	30 µm B	Hydroponics	Noguchi et al., 2000
Atbor1-1	water insoluble residues root	93%	na	100 µm B	Hydroponics	Noguchi et al., 2000
Atbor1-1	Xylem sap	36%	na	3 µm B	Hydroponics	Noguchi et al., 2000

Atbor1-1	Xylem sap	95%	na	30 µm B	Hydroponics	Noguchi et al., 2000
Atbor1-1	cell wall fractions of shoots	~ 92%	~ 94%	30 µm B	Hydroponics	Noguchi et al., 2003
Atbor1-1	cell wall fractions of shoots	~ 50%	~ 67%	3 µm B	Hydroponics	Noguchi et al., 2003
Atbor1-3	Root	~85%	~100%	0.1 µm B	agar medium	Miwa et al., 2013
Atbor2-1	Root	~100%	~83%	0.1 µm B	agar medium	Miwa et al., 2013
Atbor 2-2	Root	~100%	~83%	0.1 µm B	agar medium	Miwa et al., 2013
Atbor1-3;Atbor2-1	Root	~80%	~83%	0.1 µm B	agar medium	Miwa et al., 2013
Zmrte-1	Leaf	70% - 80%	na	2.35 ppm	Field on Molokai, HI, USA	Chatterjee et al., 2014
Zmrte-1	immature ear	~80%	na	2.35 ppm	Field on Molokai, HI, USA	Chatterjee et al., 2014
Zmrte-2	Leaf	80%	na	2.35 ppm	Field on Molokai, HI, USA	Chatterjee et al., 2014
Zmrte-2	immature ear	~50%	na	2.35 ppm	Field on Molokai, HI, USA	Chatterjee et al., 2014
Zmrte;rte2	all leaves above the top elongated internode (50 days old plants)	~61%	na	Rutgers soil + 200 µM B	Greenhouse	Chatterjee et al., 2017
Zmrte;rte2	all leaves above the top elongated internode (50 days old plants)	~77 %	na	Rutgers soil	Greenhouse	Chatterjee et al., 2017
Osbor1-1	Shoot	50%	na	Normal	Hydroponics	Nakagawa et al., 2007

Osbor1-2	Shoot	70%	na	Normal	Hydroponics	Nakagawa et al., 2007
Osbor1-2	Xylem sap	20%	na	0.03 µm B	Hydroponics	Nakagawa et al., 2007
Osbor1-2	Xylem sap	60%	na	18 µm B	Hydroponics	Nakagawa et al., 2007
Atnip5;1-2	Root	56%	na	3 µm B	Hydroponics	Takano et al., 2006
Atnip5;1-2	Shoot	22%	na	3 µm B	Hydroponics	Takano et al., 2006
Zmtls1	Roots of 2.5 week old plants	58%	na	na	Greenhouse	Durbak et al., 2014
Zmtls1	Shoots of 2.5 week old plants	76%	na	na	Greenhouse	Durbak et al., 2014
Zmtls1	4 - 5 mm developing tassels	~55% - 64%	na	na	Greenhouse	Durbak et al., 2014
Zmtls1	9 - 10 mm developing tassels	~55% - 64%	na	na	Greenhouse	Durbak et al., 2014
Zmtls1	15 - 17 mm developing tassels	~55% - 64%	na	na	Greenhouse	Durbak et al., 2014
Zmtls1	2.2 - 5.5 cm developing tassels or ears	na	70%	na	Greenhouse	Durbak et al., 2014
Zmtls1	Flagleaf	124%	na	0.39 ppm	Field in Columbia, MO, USA	Matthes et al., 2018
Zmtls1	Leaf 8	36%	na	0.08 ppm	Greenhouse	Matthes et al., 2018
Zmtls1	Leaf 8	60%	na	0.08 ppm	Greenhouse plus extra light	Matthes et al., 2018
Osdte/Osnip3;1	whole plant 30 days after transfer to B deficiency regime	~100%	na	0 µM	nutrient solution	Liu et al., 2015
Osdte/Osnip3;1	whole plant 30 days after transfer to B deficiency regime	~100%	na	0.01 µM	nutrient solution	Liu et al., 2015

Osdte/Osnip3;1	whole plant 30 days after transfer to B deficiency regime	~140%	na	0.1 µM	nutrient solution	Liu et al., 2015
Osdte/Osnip3;1	whole plant 30 days after transfer to B deficiency regime	~125%	na	1 µM	nutrient solution	Liu et al., 2015
Osdte/Osnip3;1	whole plant 30 days after transfer to B deficiency regime	~100%	na	10 µM	nutrient solution	Liu et al., 2015
Osdte/Osnip3;1	whole plant 30 days after transfer to B deficiency regime	~105%	na	100 µM	nutrient solution	Liu et al., 2015
Osdte/Osnip3;1	Root 11 days after transfer to B deficiency regime	~100%	na	0 µM	nutrient solution	Shao et al., 2018
Osdte/Osnip3;1	Shoot 11 days after transfer to B deficiency regime	~100%	na	0 µM	nutrient solution	Shao et al., 2018
Osdte/Osnip3;1	Leaf 2-7 respectively 11 days after transfer to B deficiency regime	~100%	na	0 µM	nutrient solution	Shao et al., 2018
Osdte/Osnip3;1	Root 11 days after transfer to B deficiency regime	~100%	na	3 µM	nutrient solution	Shao et al., 2018
Osdte/Osnip3;1	Shoot 11 days after transfer to B deficiency regime	~100%	na	3 µM	nutrient solution	Shao et al., 2018

Osdte/Osnip3;1	Leaf 2-7 respectively 11 days after transfer to B deficiency regime	~100%	na	3 µM	nutrient solution	Shao et al., 2018
